# Analysis of quality of life among asthmatic individuals with obesity and its relationship with pulmonary function: cross-sectional study: Análise da qualidade de vida de indivíduos asmáticos obesos e sua relação com a função pulmonar: estudo transversal

**DOI:** 10.1590/1516-3180.2016.0342250217

**Published:** 2017-07-31

**Authors:** Letícia Baltieri, Luiz Claudio Martins, Everton Cazzo, Débora Aparecida Oliveira Modena, Renata Cristina Gobato, Elaine Cristina Candido, Elinton Adami Chaim

**Affiliations:** I PT, MSc. Doctoral Student, Universidade Estadual de Campinas (UNICAMP), Campinas (SP), Brazil.; II MD, PhD. Pneumologist, Professor of Universidade Estadual de Campinas (UNICAMP), Campinas (SP), Brazil.; III MD, PhD. Attending Physician and Assistant Lecturer, Universidade Estadual de Campinas (UNICAMP), Campinas (SP), Brazil.; IV MSc. Nutritionist and Doctoral Student, Universidade Estadual de Campinas (UNICAMP), Campinas (SP), Brazil.; V MSc. Nurse and Doctoral Student, Universidade Estadual de Campinas (UNICAMP), Campinas (SP), Brazil.; VI MD, PhD. General Surgeon and Professor, Universidade Estadual de Campinas (UNICAMP), Campinas (SP), Brazil.

**Keywords:** Obesity, Asthma, Quality of life, Respiratory function tests, Spirometry

## Abstract

**CONTEXT AND OBJECTIVE:**

The combined effect of obesity and asthma may lead to significant impairment of quality of life (QOL). The aim here was to evaluate the prevalence of asthma among obese individuals, characterize the severity of impairment of quality of life and measure its relationship with pulmonary function.

**DESIGN AND SETTING:**

Observational cross-sectional study in public university hospital.

**METHODS:**

Morbidly obese individuals (body mass index > 40 kg/m^2^) seen in a bariatric surgery outpatient clinic and diagnosed with asthma, were included. Anthropometric data were collected, the Standardized Asthma Quality of Life Questionnaire (AQLQ(S)) was applied and spirometry was performed. The subjects were divided into two groups based on the median of the score in the questionnaire (worse < 4 and better ≥ 4) and were compared regarding anthropometric data and pulmonary function.

**RESULTS:**

Among the 4791 individuals evaluated, 219 were asthmatic; the prevalence of asthma was 4.57%. Of these, 91 individuals were called to start multidisciplinary follow-up during the study period, of whom 82 answered the questionnaire. The median score in the AQLQ(S) was 3.96 points and, thus, the individuals were classified as having moderate impairment of their overall QOL. When divided according to better or worse QOL, there was a statistically difference in forced expiratory flow (FEF) 25-75%, with higher values in the better QOL group.

**CONCLUSION:**

The prevalence of asthma was 4.57% and QOL was impaired among the asthmatic obese individuals. The worst QOL domain related to environmental stimuli and the best QOL domain to limitations of the activities. Worse QOL was correlated with poorer values for FEF 25-75%.

## RESUMO

**CONTEXTO E OBJETIVO:**

O efeito combinado de obesidade e asma pode levar a um comprometimento significativo da qualidade de vida (QV). O objetivo foi avaliar a prevalência de asma entre obesos, caracterizar a gravidade do comprometimento da QV e verificar sua relação com a função pulmonar.

**TIPO DE ESTUDO E LOCAL:**

Estudo transversal observacional em hospital universitário público.

**MÉTODOS:**

Foram incluídos indivíduos obesos mórbidos (indice de massa corporal > 40 kg/m^2^), acompanhados num ambulatório de cirurgia bariátrica e diagnosticados com asma. Foram coletados dados antropométricos e aplicado o Standardized Asthma Quality of Life Questionnaire (AQLQ (S)), bem como a espirometria. Os indivíduos foram divididos em dois grupos com base na mediana obtida no escore do questionário (pior < 4 e melhor ≥ 4) e os grupos foram comparados aos dados antropométricos e função pulmonar.

**RESULTADOS:**

Dos 4.791 indivíduos avaliados, 219 eram asmáticos; a prevalência de asma foi de 4,57%. Destes, 91 indivíduos foram chamados para iniciar o acompanhamento multidisciplinar no período do estudo, sendo que 82 responderam ao questionário. A pontuação mediana do AQLQ (S) foi de 3,96 pontos, portanto, classificados com prejuízo moderado na QV global. Quando divididos por melhor ou pior QV, houve diferença estatística no fluxo expiratório forçado (FEF) 25-75%, com maior valor no grupo com melhor QV.

**CONCLUSÃO:**

A prevalência da asma na população estudada foi de 4,57% e há prejuízos na QV de obesos asmáticos, sendo o pior domínio de QV relacionado aos estímulos ambientais e o melhor domínio de QV relacionado às limitações das atividades. A pior QV se relacionou a piores valores de FEF 25-75%.

## INTRODUCTION

Asthma is a chronic inflammatory disease of the airways that is associated with hyper-responsivity. It leads to recurrent episodes of wheezing, dyspnea, sensation of chest tightness and coughing, particularly at night or in the early morning. The obstruction to the airflow may be reversed spontaneously or by means of treatment. About 300 million individuals worldwide present asthma. The factors associated with the disease include environmental factors relating to allergies, occupation, smoking, infections, pollution and diet; and endogenous factors relating to genetics, gender and obesity.^1^

Asthma is diagnosed based on the symptoms and is confirmed through pulmonary function tests, such as spirometry and expiratory flow peak measurement. These enable evaluation of the severity of the limitation to the airflow and its reversibility and variability.^1^

In a meta-analysis by Beuther et al.,^2^ it was observed that obese individuals were more likely to develop asthma than were lean individuals. The exact mechanism for development of asthma is uncertain, but the inflammation mediators produced by the adipose tissue may contribute towards a low-grade systemic inflammatory state and promote changes to pulmonary function, thus leading to episodes of bronchospasm.

Today, obesity has reached epidemic levels and has become a public health concern. In 2014, more than 1.9 billion adult individuals (39%) were at least overweight, and of these, more than 600 million were obese.^3^ Obesity is defined as body mass index (BMI) greater than or equal to 30 kg/m^2^ and considered to be a multifactorial disease.^4^ Its probable causes are a combination of genetic, endocrine, behavioral, socioeconomic, psychological and environmental imbalances, and it leads to several comorbidities.^5^

Follow-up for asthmatic patients is necessary, with the aims of controlling the condition and avoiding exacerbations and the need for in-hospital assistance, especially when it is associated with obesity. Assessment of this information by means of questionnaires is useful within clinical practice and scientific research, since this allows standardization and reproducibility of measurements at low cost.

## OBJECTIVE

The aims of this study were to evaluate the prevalence of asthma in the obese population, characterize its severity of impairment of the quality of life of asthmatic obese individuals and measure its influence on pulmonary function.

## METHODS

### Study design and setting

This was an observational cross-sectional study conducted at the bariatric surgery outpatient clinic of our university’s teaching hospital. It was submitted for evaluation and was then approved by the local ethics review board (289.425). The laws and norms regarding studies on humans were followed, in accordance with resolution 196/96 of the National Health Council and all the participants in the study signed an informed consent statement.

### Sampling and participants

The power of the sample was calculated based on the global AQLQ(S) (Standardized Asthma Quality of Life Questionnaire) and a sample power of 88% was obtained.

The inclusion criteria were that the subjects needed to:

- present morbid obesity (BMI ≥ 40 kg/m^2^);
- be candidates for bariatric surgery;
- have a clinical diagnosis of asthma in accordance with the Global Initiative for Asthma consensus statement^1^ and/or antecedents of any episode of bronchospasm at any time during their lives and/or current or previous use of medication to treat asthma.

The exclusion criteria were the presence of:

- smoking habit;
- cognitive impairment that could impede performance of the clinical tests and completion of the questionnaire;
- respiratory diseases other than asthma;
- congestive heart failure or cardiovascular ischemic disease.

The recruitment period for the participants was from February 2015 to April 2016.

Adult individuals were screened at the time of registration to enter the outpatient clinic and become candidates for bariatric surgery. On this occasion, they filled out a registration form that asked for information about the presence of asthma. Those who reported having asthma or experiencing episodes of bronchospasm without an ultimate diagnosis, and who fulfilled the other criteria, were then informed about the procedures of the study and were invited to take part in it. The procedures would involve clinical confirmation of the diagnosis of asthma by means of consultations with a physiotherapist, evaluation with a pneumologist physician and performance of spirometry.

### Pulmonary function tests and asthma diagnosis

Asthma was investigated based on the symptoms that individuals reported having had over their whole lifetime, such as episodes of bronchospasm, breathlessness, sensation of chest tightness and coughing,^1^^,^^6^ or in situations in which individuals were routinely using medications for asthma, in accordance with the Global Initiative for Asthma consensus statement.^1^ Once diagnosed, these individuals would undergo pulmonary function tests to assess the severity of the disease. Other respiratory diseases were excluded based on anamnesis and pulmonary function test.

Spirometry was performed at the Pulmonary Function Laboratory under supervision by a technical team and the norms of the American Thoracic Society (ATS) and European Respiratory Society (ERS)^7^ were followed. To evaluate measurements of pulmonary volumes and flows, two maneuvers were performed: slow vital capacity and forced vital capacity. The maneuvers were performed repeatedly until three acceptable curves were obtained, of which two needed to be reproducible. The total number of trials could not exceed eight. The subjects rested for 10 minutes before the test and received appropriate orientations during the test.

The maneuvers were performed at two times: before and after using a bronchodilator (salbutamol, 200-400 µg) to observe the increase in the forced expiratory volume in the first second (FEV_1_) and/or the peak expiratory flow (PEF). Asthma is diagnosed when there is a 12% or 200 ml increase in FEV_1_ and a 20% or 60 liter/min increase in PEF, in relation to the pre-bronchodilator values. The subjects were instructed to suspend their use of bronchodilator for 8-12 hours before the test.^1^^,^^6^

### Evaluation procedures and outcome measurements

Antropometric data were collected and the quality of life was assessed follows.

The following anthropometric data were collected: weight, height and BMI. Weight was measured by means of a digital weighing machine (Filizola ID-1500, Brazil), with a capacity of 300 kg capacity and precision of 0.1 kg. Height was measured by means of a wall-mounted stadiometer, with a capacity of 2 meters and precision of 0.1 cm. Body mass index (BMI) was calculated by means of Quetelet’s formula,^8^ i.e. weight/(height^2^).

Quality of life was assessed by means of the Standardized Asthma Quality of Life Questionnaire (AQLQ(S)), which is a self-applicable questionnaire consisting of 32 questions that evaluate the last two weeks within four separate domains (impairment of activities, symptoms, emotions and environmental stimuli). It was developed by Juniper et al.^9^ and was validated and standardized by Juniper et al.^10^ It has been translated into Portuguese for use in Brazil, as well as into more than 30 other languages. The Brazilian Portuguese version was validated and was considered to have good reproducibility and characteristics similar to those of the original instrument.^11^ Thus, it could be used for the population of the present study.

The questionnaire scores are calculated from the means of each domain; the scores range from 1 to 7. The higher the score is, the better the quality of life is. The questionnaire contains specific questions relating to asthma and respiratory symptoms that are triggered in specific activities and, therefore, assesses these conditions without connection with obesity.

### Statistical analysis

The data were encoded for the SPSS 13.0 software and descriptive analysis was performed. The individuals were divided into two groups based on the scores obtained from the questionnaire (better or worse quality of life). The cutoff value for defining the groups was obtained through descriptive analysis on the overall AQLQ(S), which found a median score of 3.96 points. Thus, the cutoff value of 4 was used. In addition, according to Juniper et al.,^9^ 4.0 is an intermediate score in the questionnaire and therefore separates between worse and better quality of life.

In this manner, the subjects were then divided between group 1 (worse QOL; score < 4) and group 2 (better QOL; score ≥ 4). These groups were compared regarding their anthropometric data and pulmonary function results, by means of the Mann-Whitney test. The significance level used was 5% (P-value < 0.05).

## RESULTS

**Figure 1.**
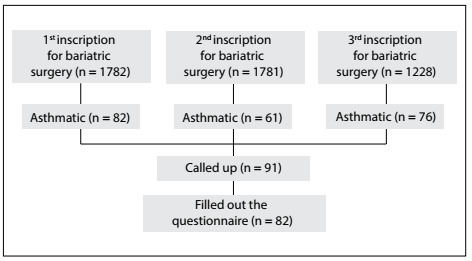
Patient recruitment flow in the study.

During the study period, there were three inscription events to enlist candidates for bariatric surgery at our service. On the first occasion (March 2014), 1,782 individuals were registered and, of these, 82 (4.6%) were reported as asthmatic; on the second occasion (December 2014), 1,781 were registered and, of these, 61 (3.42%) were reported as asthmatic; and on the third occasion (November, 2015), 1,228 individuals were registered and, of these, 76 (6.18%) were reported as asthmatic. Hence, out of an overall population of 4,791 individuals with obesity, 219 (5.57%) reported having asthma symptoms. Figure 1 shows a graphic representation of this phase of the recruitment.

For patients to be called up to begin the preoperative program, the criteria used were their severity of obesity and comorbidities, their position on the waiting list and the availability of preoperative examinations and surgical vacancies. Up to the end of the study period, 91 asthmatic individuals were called up to enter the program and, of these, 82 adequately filled out the proposed questionnaire. All the individuals in our sample who reported in this file that they had suggestive symptoms and who were called up for the program were confirmed as having a clinical diagnosis of asthma.

Anthropometric data were collected from these 82 individuals and are presented in Table 1. After analysis on the sample, they were stratified as having “better” or “worse” QOL, according to the scores obtained in the questionnaire. The features that significantly differed between the groups were height (P = 0.004) and pre-bronchodilator pulmonary function test values, which presented a significant difference regarding forced expiratory flow (FEF) 25-75%, which was higher in the group with better QOL group (P = 0.043). Table 2 shows the characteristics of both groups.

**Table 1.**
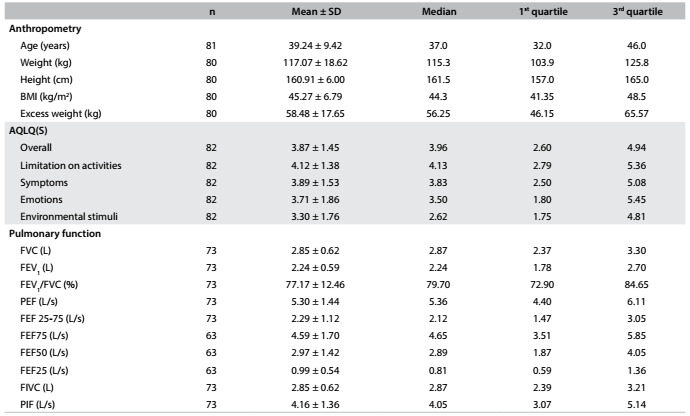
Overall characteristics of the study population. Data expressed as means and standard deviations (SD), and as medians and quartiles

BMI = body mass index; AQLQ(S) = Standardized Asthma Quality of Life Questionnaire*;* FVC = forced vital capacity; FEV_1_ = forced expiratory volume in 1^st^ second; PEF = peak expiratory flow; FEF = forced expiratory flow; FIVC = forced inspiratory vital capacity; PIF = peak inspiratory flow; L = liter.

**Table 2.**
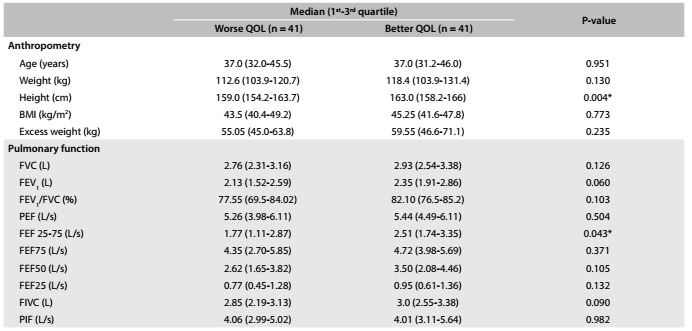
Comparison of the pre-bronchodilator pulmonary function tests between the “better” and “worse” quality of life (QOL) groups (cutoff value = 4)

*Statistically significant P value; BMI = body mass index; AQLQ(S) = Standardized Asthma Quality of Life Questionnaire; FVC = forced vital capacity; FEV_1_ = forced expiratory volume in 1^st^ second; PEF = peak expiratory flow; FEF = forced expiratory flow; FIVC = forced inspiratory vital capacity; PIF =: peak inspiratory flow; L = liter.

## DISCUSSION

In a meta-analysis conducted by Beuther et al.,^2^ obese individuals presented higher risk of developing asthma than did lean subjects. The prevalence of asthma in the present study was 4.57% in a population of 4,791 individuals. This prevalence is low in comparison with what was found in the study by Melo et al.,^12^ which was 18.5% in a population of 363 obese individuals. However, in our study, asthma was reported in our subjects’ registration files for their entry to the preoperative program for bariatric surgery, i.e. before contact with the multiprofessional team or detailed clinical interview.

It is known that asthma may be underdiagnosed in low-income obese populations for several reasons, such as poor access to information or to specific healthcare services that provide diagnosis and management of asthma. Moreover, individuals may interpret their own episodes of wheezing as physical tiredness caused by obesity, which would remit without use of medications or medical evaluation. In such situations, they might not provide this information at the time that the registration file is filled out. However, all the individuals in our sample who reported in this file that they had suggestive symptoms and who were called up for the program were confirmed as having a clinical diagnosis of asthma, determined through the reported clinical history.

All the individuals in this study present grade III obesity (BMI 40-49.9 kg/m^2^), with a mean BMI of 45.27 ± 6.79 kg/m^2^. Grade III obesity causes severe changes to pulmonary function due to several factors, such as fat deposition around the thorax and abdomen, which limits adequate movements of the thorax^13^ and changes pulmonary compliance.^14^^,^^15^ This leads to microatelectasis in the pulmonary inferior lobes^16^^,^^17^ and reduces functional capacity,^13^^,^^18^ which compromises performance of simple daily activities, due to early tiredness. Furthermore, the low-grade systemic inflammatory state caused by fat tissue has the capacity to influence the lung parenchyma,^19^ thereby leading to episodes of bronchospasm.

Besides changes to pulmonary function, obesity may lead to physical limitations, postural changes and joint overload,^20^ which gives rise to joint pain and impairment of walking ability and daily activities. Such impairments, both pulmonary and physical, directly affect the QOL of these individuals, and weight loss is strongly recommended. Hence, the individuals called up for the study were instructed to begin preparations for the preoperative assessment for bariatric surgery, which favors a healthy lifestyle, especially regarding diet and physical activity.

The QOL data obtained demonstrated that the individuals scored in the medium band of the score scale from 1-7 (median = 3.96 points) and, thus, presented moderately compromised QOL in all the domains evaluated. The domain with the best final score related to limitations on activities (median = 4.13 points) and the worst related to environmental stimuli (median = 2.62 points).

The domain with the best score (albeit still denoting moderate impairment), relating to limitations on activities, comprised questions on specific daily activities that may cause episodes of bronchospasm and breathlessness and the degree of limitation that these cause to the individual (such as walking, running, practicing exercises, working, socializing etc.). These were not necessarily physical limitations, but could also be limitations relating to fear of exposure to risky situations.

The worst-scoring domain related to environmental stimuli, which comprised specific questions on symptoms caused by smoke, dust, foul weather, pollution and perfume fragrances. External environmental stimuli may potentiate systemic pulmonary inflammation, thus leading to hyperresponsivity of the airways and episodes of bronchospasm.

When the individuals were stratified into two groups according to their asthma-related QOL, it was observed that the individuals with better QOL also presented significantly higher FEF 25-75% (P = 0.043). FEF represents the mean forced expiratory flow in the intermediate band of forced vital capacity (FVC), i.e. between 25 and 75% of the FVC curve.^21^ FEF 25-75% depends on the elastic retraction force of the lungs, the permeability of the small airways and the muscle strength. Its measurement provides information on the permeability of the small airways and is unrelated to the patient’s collaboration.^22^ Thus, all of the mechanical and inflammatory changes present in the lungs of morbidly obese individuals may lead to changes in the permeability of low-caliber airways, which is mirrored in measurements of FEF 25-75%.^12^

Although the FEV_1_/FVC% ratio is the measurement that best represents obstructive disorders,^21^ it was normal in our study, albeit at the lower limit. According to Pereira,^21^ patients with established chronic obstructive pulmonary disease (COPD) tend to show much more surprising changes in FEF 25-75% than in the FEV_1_/FVC% ratio. However, because of the correlation between FEF 25-75% and FEV_1_/FVC%, the FEF 25-75% measurement becomes redundant when the FEV_1_/FVC% ratio is abnormal. Therefore, if the FEV_1_/FVC ratio is borderline, a reduction in FEF 25-75% or other terminal flows indicates airflow obstruction in individuals with symptomatic respiratory disorders.

According to Lebecque et al.,^22^ for mild asthma, FEF 25-75% appeared to be more sensitive than the FEV_1_/FVC ratio for indicating the presence of small-caliber airway obstruction.

In the present study, although no relationship was found for other spirometric variables, it could be seen that the values of FEV_1_, FEF and FEF 25% also were below the normal range when the non-stratified sample was analyzed, this finding is expected in asthmatic individuals.^21^^,^^23^

Some studies in which pulmonary function tests were performed on obese individuals without pulmonary abnormalities showed significant reductions in functional residual capacity (FRC)^13^^,^^24^ and expiratory reserve volume (ERV)^13^^,^^25^^,^^26^ that were attributable to the mechanical changes that fat tissue causes to the thorax. Nonetheless, the changes in FEF 25-75% was attributed by Sood^13^ to inflammatory changes that occurred in the lungs of obese individuals, thereby leading to premature closure of the small airways during forced expiration. This might explain the relationship between the severity of asthma and the observed values of FEF 25-75%. Such changes in the small airways may contribute towards situations in which low effort or low environmental stimuli provoke episodes of bronchospasm and breathlessness, thus compromising the QOL of these individuals.

There is recent evidence highlighting the burdens on QOL caused by certain situations, such as asthma. In a study that used the same QOL evaluation questionnaire as in the present study, Rocha^27^ observed that asthma had a significant impact on QOL even when partly controlled. Furthermore, in a review of literature conducted by Araújo et al.,^28^ it was concluded that the QOL and sleep quality of asthmatic individuals were compromised. On the other hand, in a study by Pereira et al.^29^ that used the Saint George’s Respiratory Questionnaire (SGRQ) to assess the QOL of individuals with asthma and chronic obstructive pulmonary disease (COPD), it was observed that, when the disease was classified as mild to moderate and was adequately treated, there was no impairment of QOL.

In studies that evaluated the impact of obesity on QOL, there was evidence that obesity led to impairment of QOL. Weight loss might improve the overall QOL within this group.^30^^,^^31^^,^^32^

Hence, since impairments of QOL occur in both diseases, an association between them would be expected to cause even more damage. This explains the importance of measuring QOL in these cases, in such a way that therapeutic strategies and goals can be designed.

Since we identified that worse QOL in the present study was related to greater impairment of pulmonary function, it is possible for the attending physician to identify individuals with disease of greater severity by means of a simple questionnaire that may be self-applicable. This would reduce the need for additional pulmonary function tests and, thus, minimize the cost of therapy for these individuals, since improvement of the symptoms and QOL should be the ultimate goal.

Therefore, the possibility of classifying the QOL of asthmatic obese individuals by means of a questionnaire may provide attending physicians with significant information on the degree of impairment of pulmonary function in these individuals and make it possible to define strategies for better and individualized therapy.

### Limitations

Although the Brazilian Portuguese version of the questionnaire has many properties similar to the original instrument, and is a valid instrument for this population according to the authors who validated it, these authors mentioned in their validation study that hardly any study can claim to provide full validation. Therefore, studies that validate the questionnaire more appropriately would be required, and this might constitute a form of bias for research that uses the instrument. Nonetheless, the original questionnaire was translated into Brazilian Portuguese in accordance the internationally accepted methodology.

## CONCLUSION

The prevalence of asthma in the study population was 4.57%. The QOL of individuals with asthma and obesity was impaired. The worst QOL domain related to environmental stimuli and the best QOL domain related to the limitations of the activities. Worse QOL correlated with lower values for FEF 25-75% in the pulmonary function test.

## References

[1] Global Initiative for Asthma (2017). Global strategy for asthma management and prevention.

[2] Beuther DA, Sutherland ER (2007). Overweight, obesity, and incident asthma: a meta-analysis of prospective epidemiologic studies. Am J Respir Crit Care Med.

[3] World Health Organization. Media centre Obesity and overweight.

[4] World Health Organization (2003). Global strategy on diet, physical activity and health. World Health Organization.

[5] Yurcisin BM, Gaddor MM, DeMaria EJ (2009). Obesity and bariatric surgery. Clin Chest Med.

[6] Sociedade Brasileira de Pneumologia e Tisiologia (2012). Diretrizes da Sociedade Brasileira de Pneumologia e Tisiologia para o Manejo da Asma - 2012. Jornal Brasileiro de Pneumologia.

[7] Miller MR, Hankinson J, Brusasco V (2005). Standardisation of spirometry. Eur Respir J.

[8] Quetelet AD (1871). Antropométrie ou Mésure des Différences Facultés de l’Homme.

[9] Juniper EF, Guyatt GH, Epstein RS (1992). Evaluation of impairment of health related quality of life in asthma: development of a questionnaire for use in clinical trials. Thorax.

[10] Juniper EF, Buist AS, Cox FM, Ferrie PJ, King DR (1999). Validation of a standardized version of the Asthma Quality of Life Questionnaire. Chest.

[11] Silva LMC, Silva LCC (2007). Validação do questionário de qualidade de vida em asma (Juniper) para o português brasileiro [Validation of asthma quality of life questionnaire (Juniper) to brazilian portuguese]. Revista da AMRIGS.

[12] Melo SMD, Alves AJ, Menezes RS, Melo VA (2011). Prevalência e gravidade de asma brônquica em adultos obesos com indicação de cirurgia bariátrica [Prevalence and severity of asthma in obese adult candidates for bariatric surgery]. J Bras Pneumol.

[13] Sood A (2009). Altered resting and exercise respiratory physiology in obesity. Clin Chest Med.

[14] Dumont L, Mattys M, Mardirosoff C (1997). Changes in pulmonary mechanics during laparoscopic gastroplasty in morbidly obese patients. Acta Anaesthesiol Scand.

[15] Pelosi P, Croci M, Calappi E (1996). Prone positioning improves pulmonary function in obese patients during general anesthesia. Anesth Analg.

[16] Eichenberger AS, Proietti S, Wicky S (2002). Morbid obesity and postoperative pulmonary atelectasis: an underestimated problem. Anesth Analg.

[17] Baltieri L, Peixoto-Souza FS, Rasera-Junior I (2016). Análise da prevalência de atelectasia em pacientes submetidos à cirurgia bariátrica [Analysis of the prevalence of atelectasis in patients undergoing bariatric surgery]. Rev Bras Anestesiol.

[18] McCallister JW, Adkins EJ, O’Brien JM (2009). Obesity and acute lung injury. Clin Chest Med.

[19] Thyagarajan B, Jacobs DR, Smith LJ (2010). Serum adiponectin is positively associated with lung function in young adults, independent of obesity: the CARDIA study. Respir Res.

[20] Toivanen AT, Heliövaara M, Impivaara O (2010). Obesity, physically demanding work and traumatic knee injury are major risk factors for knee osteoarthritis--a population-based study with a follow-up of 22 years. Rheumatology (Oxford).

[21] Pereira CAC (2002). Espirometria. J Pneumol.

[22] Lebecque P, Kiakulanda P, Coates AL (1993). Spirometry in the asthmatic child: is FEF25-75 a more sensitive test than FEV1/FVC?. Pediatr Pulmonol.

[23] Mallozi MC, Rozov T (1998). O laboratório nas doenças pulmonares [Laboratorial tests in pulmonary diseases]. J Pediatr (Rio J.).

[24] Guimarães C, Martins MV, Moutinho dos Santos J (2012). Função pulmonar em doentes obesos submetidos a cirurgia bariátrica. Revista Portuguesa de Pneumologia.

[25] Rasslan Z, Saad R, Stirbulov R, Fabbri RMA, Lima CAC (2004). Avaliação da função pulmonar na obesidade graus I e II [Evaluation of pulmonary function in class I and II obesity]. J Bras Pneumol.

[26] Baltieri L, Santos LA, Pazzianotto-Forti EM, Montebelo MIL, Rasera-Junior I (2014). Uso da pressão positiva em cirurgia bariátrica e efeitos sobre a função pulmonar e prevalência de atelectasias: estudo randomizado e cego [Use of positive pressure in the bariatric surgery and effects on pulmonary function and prevalence of atelectasis: randomized and blinded clinical trial]. ABCD Arq Bras Cir Dig.

[27] Rocha CC (2013). Qualidade de vida e inflamação das vias aéreas em diferentes níveis de controle da asma [dissertation].

[28] Araújo DL, Souza-Machado A, Souza-Machado C, Salles C (2014). Avaliação da qualidade do sono e da qualidade de vida na asma [Evaluation of quality of sleep and quality of life in asthma]. Braz J Allergy Immunol.

[29] Pereira EA, Ferreira PR, Araújo MEA, Carvalho STRF, Carvalho LN (2016). Estudo comparativo da qualidade de vida entre pacientes com doença pulmonar obstrutiva crônica e pacientes asmáticos. Revista Ceuma Perspectivas.

[30] Pimenta GP, Jaudy TR, Moura DN (2013). Avaliação da qualidade de vida tardia após gastroplastia vertical [Long-term quality of life after vertical sleeve gastroplasty]. Rev Col Bras Cir.

[31] Barros LM, Moreira RAN, Frota NM, Araújo TM, Caetano JA (2015). Qualidade de vida entre obesos mórbidos e pacientes submetidos à cirurgia bariátrica [Quality of life among morbid obese and patients submitted to bariatric surgery]. Revista Eletrônica de Enfermagem.

[32] Gomes T, Teixeira RAT, Nascimento DC (2015). Qualidade de vida e síndrome metabólica em mulheres brasileiras: análise da correlação com a aptidão aeróbia e a força muscular [Quality of life and metabolic syndrome in Brazilian women: analysis of the correlation with aerobic fitness and muscle strength]. Motri.

